# Saint Thomas's Hospital Reports

**Published:** 1892-12

**Authors:** 


*Saint Thomas's Hospital Reports.* Edited by Dr. HADDEN and Mr. ANDERSON. Vol. XX. Pp. xxxvi., 483. London: J. & A. CHURCHILL. 1892.

This volume has a melancholy preface, containing memorial notices of four men who accomplished much for their profession and the school to which they were attached. Thomas Alfred Barker and Sir James Risdon Bennett died at 84 and 82 respectively, whilst William Henry Stone and Alfred James Bernays died at the earlier ages of 61 and 69. These obituary notices, by their friends J. S. B. and W. M. O., must be of the deepest interest to all old St. Thomas's students, and to a much larger world outside.

The papers forming the body of the book are of the usual standard of excellence. That by Dr. Hawkins "On Tubercular Peritonitis" appears to show that the physicians may even yet claim the privilege of guiding many cases to a successful issue without the active assistance of a surgical colleague. An interesting paper "On Specific Diseases," by Dr. Joseph Frank Payne, deals with the subject from the point of view of the bacterium, and concludes in the following words; "The combat remains with these subtle and pervading enemies, more terrible and fatal than the tiger or the serpent; and in this combat the first essential is to know thoroughly the laws governing that life which is antagonistic to our own."

